# *catena*-Poly[[bis­(pyridine-κ*N*)cadmium]-di-μ_2_-thio­cyanato-κ^2^ *N*:*S*;κ^2^ *S*:*N*]

**DOI:** 10.1107/S1600536811009871

**Published:** 2011-03-26

**Authors:** Jan Boeckmann, Inke Jess, Christian Näther

**Affiliations:** a Institut für Anorganische Chemie, Christian-Albrechts-Universität Kiel, Max-Eyth Strasse 2, D-24098 Kiel, Germany

## Abstract

The asymmetric unit of the title compound, [Cd(NCS)_2_(C_5_H_5_N)_2_]_*n*_, consists of two crystallographically independent Cd^II^ cations, four thio­cyanato anions and four pyridine ligands. The Cd^II^ atoms are each coordinated by four N atoms from two pyridine ligands and two thio­cyanato anions, each in a mutually *cis* orientation, and by two S atoms from two adjacent thio­cyanato anions within a slightly distorted octa­hedral coordination environment. The Cd^II^ atoms are μ-1,3-bridged *via* the thio­cyanato anions into polymeric chains parallel to [001]. The Cd^II^⋯Cd^II^ intra­chain separations range between 5.9688 (6) and 6.0195 (6) Å, whereas the shortest Cd^II^⋯Cd^II^ inter­chain separations are 7.8272 (7) and 8.6312 (6) Å.

## Related literature

For related structures see: Boeckmann & Näther (2010 ▶); Chen *et al.* (2005 ▶ **)**; Foner *et al.* (1975 ▶); Marsh *et al.* (2002 ▶); Porai-Koshits & Tishchenko (1960 ▶); Reller & Oswald (1986 ▶); Taniguchi *et al.* (1987 ▶); Zhu *et al.* (2008 ▶).
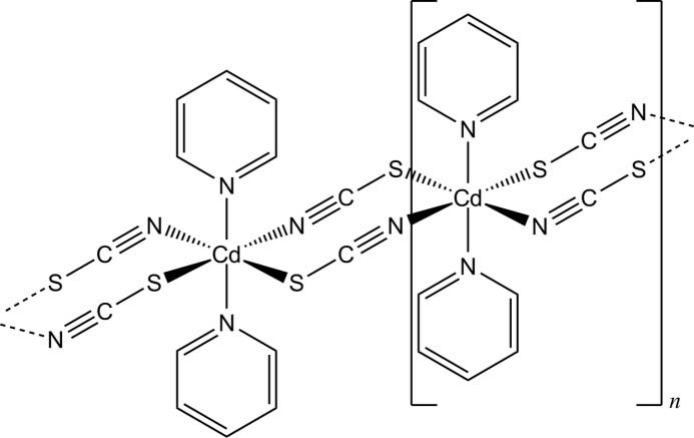


## Experimental

### 

#### Crystal data

[Cd(NCS)_2_(C_5_H_5_N)_2_]
*M* *_r_* = 386.76
Triclinic, 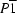

*a* = 7.8272 (4) Å
*b* = 8.6242 (4) Å
*c* = 23.705 (1) Å
α = 84.890 (3)°
β = 89.520 (4)°
γ = 63.070 (3)°
*V* = 1420.06 (11) Å^3^
*Z* = 4
Mo *K*α radiation
μ = 1.82 mm^−1^
*T* = 293 K
0.15 × 0.11 × 0.07 mm

#### Data collection

Stoe IPDS-2 diffractometer
Absorption correction: numerical (*X-SHAPE* and *X-RED32*; Stoe & Cie, 2008) ▶ *T*_min_ = 0.779, *T*_max_ = 0.874
21468 measured reflections
5998 independent reflections
4613 reflections with *I* > 2σ(*I*)
*R*_int_ = 0.071

#### Refinement

*R*[*F* ^2^ > 2σ(*F* ^2^)] = 0.052
*wR*(*F* ^2^) = 0.083
*S* = 1.17
5998 reflections
343 parameters
H-atom parameters constrained
Δρ_max_ = 0.60 e Å^−3^
Δρ_min_ = −0.75 e Å^−3^

### 

Data collection: *X-AREA* (Stoe & Cie, 2008) ▶; cell refinement: *X-AREA* ▶; data reduction: *X-AREA* ▶; program(s) used to solve structure: *SHELXS97* (Sheldrick, 2008 ▶); program(s) used to refine structure: *SHELXL97* (Sheldrick, 2008 ▶); molecular graphics: *XP* in *SHELXTL* (Sheldrick, 2008 ▶) and *DIAMOND* (Brandenburg, 2011 ▶); software used to prepare material for publication: *SHELXL97*.

## Supplementary Material

Crystal structure: contains datablocks I, global. DOI: 10.1107/S1600536811009871/bt5493sup1.cif

Structure factors: contains datablocks I. DOI: 10.1107/S1600536811009871/bt5493Isup2.hkl

Additional supplementary materials:  crystallographic information; 3D view; checkCIF report

## Figures and Tables

**Table 1:** Selected bond angles (°)

N1—C1—S1	178.5 (4)
N3—C3—S3	179.1 (5)
N2—C2—S2	179.7 (5)
N4—C4—S4	178.8 (5)

**Fig. 1.**
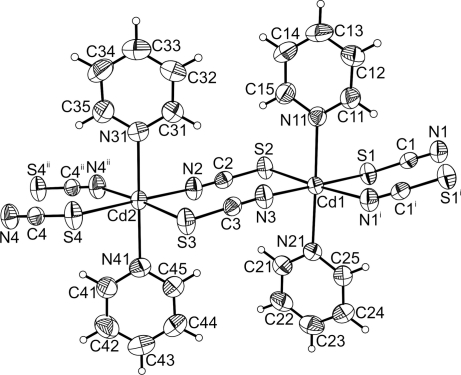
: Crystal structure of the title compund with labelling and displacement ellipsoids drawn at the 50% probability level. Symmetry codes: i = -x + 1, -y + 2, -z + 1; ii = -x + 1, -y + 2, -z.

**Fig. 2.**
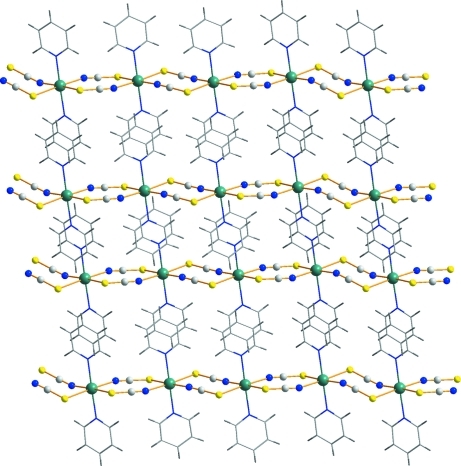
: Packing diagram of the title compound with view along the crystallographic a axis onto the one-dimensional polymeric chains.

**Fig. 3.**
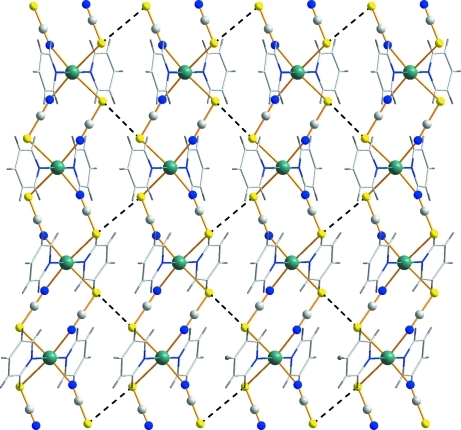
: Packing diagram of the title compound with view along the crystallographic b axis onto the layers located in the ac plane.

## supplementary crystallographic information

### Comment

The structure determination of the title compound was performed as a part of a project on the synthesis of new one-dimensional coordination compounds (Boeckmann & Näther, 2010). Within this project we have reacted cadmium(II)chloride with potassium(I)thiocyanate and pyridine in water, which leads to the phase pure formation of *catena*-poly[bis(µ_2_-thiocyanato-*N*, *S*)-bis(pyridine-*N*)cadmium(II)].

The title compound crystallizes in the centrosymmetric triclinic space group *P*1 with four formula units in the unit cell. In the crystal structure each of the two crystallographically independent cadmium atoms is surrounded by four N-atoms of two pyridine ligands and two  thiocyanato anions, each in mutually *cis* orientation, and by two S-atoms of two adjacent thiocyanato anions in a slightly distorted octahedral geometry (Fig. 1 and Tab. 1). The thiocyanato anions bridge the metal cations forming one-dimensional chains (Fig. 2), which elongate along the crystallographic *c* axis. These chains are arranged in a staggered form and further linked by weak S···S interactions into layers which are located in the *ac* plane (Fig. 3). A compound of similar composition [Cd(NCS)_2_(pyridine)_2_]*_n_* has already been described (Taniguchi *et al.*, 1987) and reported to crystallize in the centrosymmetric triclinic space group *P*1 with six formula units in the unit cell. However, Marsh *et al.* (2002) found that the triclinic cell can be transformed to a C-centered monoclinic cell with *Z* = 12. It must be noted that in both cases only unit-cell parameters but no atomic coordinates are reported. Similiar one-dimensional coordination polymers with different transition metals have also been reported (Chen *et al.* (2005); Foner *et al.* (1975); Porai-Koshits & Tishchenko (1960); Reller & Oswald (1986); Zhu *et al.* (2008).

### Experimental

The title compound was prepared by the reaction of 91.60 mg CdCl_2_ (0.50 mmol), 97.2 mg KSCN (1.00 mmol) and 20.2 µ*L* pyridine (0.25 mmol) in 1.50 ml water at RT in a closed 3 ml snap cap vial. After one week colourless needles of the title compound were obtained.

### Refinement

All H atoms were located in difference map but were positioned with idealized geometry and were refined  with *U*_eq_(H) = 1.2 *U*_eq_(C) using a riding model with C—H = 0.93 Å. The triclinic unit cell of the title compound can be transformed into a monoclinic C-centered cell but the internal *R*-value of 0.271 clearly indicates that the crystal symmetry is triclinic. We also checked our model for higher symmetry using *PLATON* but without success. However, the structure can be solved in space group C2 but refinement leads to very poor reliability factors and severe disorder. Finally ee also have checked if the crystal is pseudo-merohedrically twinned, which is not the case.

### Crystal data

**Table:**

[Cd(NCS)_2_(C_5_H_5_N)_2_]	*Z* = 4
*M**_r_* = 386.76	*F*(000) = 760
Triclinic, *P*1	*D*_x_ = 1.809 Mg m^−^^3^
Hall symbol: -P 1	Mo *K*α radiation, λ = 0.71073 Å
*a* = 7.8272 (4) Å	Cell parameters from 21468 reflections
*b* = 8.6242 (4) Å	θ = 1.7–26.8°
*c* = 23.705 (1) Å	µ = 1.82 mm^−^^1^
α = 84.890 (3)°	*T* = 293 K
β = 89.520 (4)°	Needle, colourless
γ = 63.070 (3)°	0.15 × 0.11 × 0.07 mm
*V* = 1420.06 (11) Å^3^	

### Data collection

**Table:**

Stoe IPDS-2 diffractometer	5998 independent reflections
Radiation source: fine-focus sealed tube	4613 reflections with *I* > 2σ(*I*)
graphite	*R*_int_ = 0.071
ω scans	θ_max_ = 26.8°, θ_min_ = 1.7°
Absorption correction: numerical (*X-SHAPE* and *X-RED32*; Stoe & Cie, 2008)	*h* = −9→9
*T*_min_ = 0.779, *T*_max_ = 0.874	*k* = −10→10
21468 measured reflections	*l* = −29→29

### Refinement

**Table:**

Refinement on *F*^2^	Primary atom site location: structure-invariant direct methods
Least-squares matrix: full	Secondary atom site location: difference Fourier map
*R*[*F*^2^ > 2σ(*F*^2^)] = 0.052	Hydrogen site location: inferred from neighbouring sites
*wR*(*F*^2^) = 0.083	H-atom parameters constrained
*S* = 1.17	*w* = 1/[σ^2^(*F*_o_^2^) + (0.0176*P*)^2^ + 0.9412*P*] where *P* = (*F*_o_^2^ + 2*F*_c_^2^)/3
5998 reflections	(Δ/σ)_max_ < 0.001
343 parameters	Δρ_max_ = 0.60 e Å^−^^3^
0 restraints	Δρ_min_ = −0.75 e Å^−^^3^

### Special details

**Table:**

Geometry. All e.s.d.'s (except the e.s.d. in the dihedral angle between two l.s. planes) are estimated using the full covariance matrix. The cell e.s.d.'s are taken into account individually in the estimation of e.s.d.'s in distances, angles and torsion angles; correlations between e.s.d.'s in cell parameters are only used when they are defined by crystal symmetry. An approximate (isotropic) treatment of cell e.s.d.'s is used for estimating e.s.d.'s involving l.s. planes.
Refinement. Refinement of *F*^2^ against ALL reflections. The weighted *R*-factor *wR* and goodness of fit *S* are based on *F*^2^, conventional *R*-factors *R* are based on *F*, with *F* set to zero for negative *F*^2^. The threshold expression of *F*^2^ > σ(*F*^2^) is used only for calculating *R*-factors(gt) *etc*. and is not relevant to the choice of reflections for refinement. *R*-factors based on *F*^2^ are statistically about twice as large as those based on *F*, and *R*- factors based on ALL data will be even larger.

### Fractional atomic coordinates and isotropic or equivalent isotropic displacement parameters (Å^2^)

**Table:**

	*x*	*y*	*z*	*U*_iso_*/*U*_eq_	
Cd1	0.55905 (5)	0.97050 (4)	0.375033 (15)	0.03786 (11)	
Cd2	0.44978 (6)	0.99144 (5)	0.125075 (15)	0.03970 (11)	
S1	0.8352 (2)	0.93622 (19)	0.45431 (6)	0.0470 (3)	
S3	0.2004 (2)	0.9601 (2)	0.20176 (6)	0.0542 (4)	
S2	0.8141 (2)	0.9986 (2)	0.29930 (6)	0.0479 (3)	
S4	0.1714 (2)	1.0203 (2)	0.04890 (7)	0.0542 (4)	
N1	0.6266 (7)	1.0543 (6)	0.5514 (2)	0.0483 (11)	
N3	0.3481 (7)	0.9946 (6)	0.3040 (2)	0.0501 (12)	
N2	0.6584 (7)	0.9716 (6)	0.1968 (2)	0.0512 (12)	
N11	0.4113 (6)	1.2780 (5)	0.37531 (19)	0.0424 (10)	
N21	0.7184 (7)	0.6634 (5)	0.3729 (2)	0.0452 (11)	
N31	0.2874 (7)	1.2985 (6)	0.1265 (2)	0.0494 (12)	
N41	0.5944 (7)	0.6842 (6)	0.1253 (2)	0.0511 (12)	
C1	0.7117 (7)	1.0078 (6)	0.5111 (2)	0.0371 (11)	
C3	0.2885 (7)	0.9809 (6)	0.2618 (2)	0.0380 (11)	
C2	0.7225 (7)	0.9828 (6)	0.2393 (2)	0.0382 (11)	
C4	0.2834 (8)	1.0002 (6)	−0.0110 (2)	0.0386 (11)	
C11	0.3248 (9)	1.3573 (7)	0.4204 (3)	0.0548 (15)	
H11	0.3199	1.2890	0.4524	0.066*	
C13	0.2505 (10)	1.6376 (7)	0.3758 (3)	0.0653 (18)	
H13	0.1991	1.7580	0.3764	0.078*	
C14	0.3353 (10)	1.5601 (8)	0.3289 (3)	0.0614 (17)	
H14	0.3396	1.6272	0.2964	0.074*	
C15	0.4150 (9)	1.3809 (7)	0.3299 (3)	0.0538 (14)	
H15	0.4738	1.3290	0.2976	0.065*	
C21	0.8169 (9)	0.5864 (7)	0.3292 (3)	0.0543 (15)	
H21	0.8185	0.6566	0.2972	0.065*	
C22	0.9167 (10)	0.4081 (8)	0.3286 (3)	0.0624 (17)	
H22	0.9840	0.3597	0.2969	0.075*	
C23	0.9157 (9)	0.3043 (8)	0.3745 (3)	0.0623 (17)	
H23	0.9842	0.1835	0.3751	0.075*	
C24	0.8136 (11)	0.3782 (8)	0.4199 (3)	0.0664 (18)	
H24	0.8091	0.3092	0.4518	0.080*	
C25	0.7165 (10)	0.5588 (7)	0.4175 (3)	0.0561 (15)	
H25	0.6466	0.6094	0.4486	0.067*	
C31	0.2529 (10)	1.3725 (8)	0.1749 (3)	0.0601 (16)	
H31	0.3017	1.3017	0.2086	0.072*	
C33	0.0767 (10)	1.6554 (8)	0.1281 (4)	0.0691 (19)	
H33	0.0063	1.7757	0.1287	0.083*	
C34	0.1113 (11)	1.5804 (8)	0.0782 (3)	0.075 (2)	
H34	0.0647	1.6488	0.0439	0.090*	
C35	0.2159 (10)	1.4025 (8)	0.0794 (3)	0.0636 (17)	
H35	0.2377	1.3525	0.0453	0.076*	
C41	0.6102 (10)	0.6087 (8)	0.0777 (3)	0.0619 (17)	
H41	0.5715	0.6788	0.0435	0.074*	
C42	0.6816 (11)	0.4304 (9)	0.0770 (3)	0.074 (2)	
H42	0.6885	0.3819	0.0430	0.088*	
C43	0.7418 (10)	0.3267 (8)	0.1266 (4)	0.0678 (19)	
H43	0.7921	0.2061	0.1270	0.081*	
C44	0.7273 (12)	0.4020 (9)	0.1756 (4)	0.075 (2)	
H44	0.7653	0.3340	0.2102	0.090*	
C45	0.6555 (10)	0.5800 (8)	0.1730 (3)	0.0624 (17)	
H45	0.6494	0.6303	0.2066	0.075*	
C32	0.1479 (12)	1.5493 (8)	0.1769 (4)	0.074 (2)	
H32	0.1254	1.5965	0.2116	0.088*	
C12	0.2420 (10)	1.5359 (8)	0.4219 (3)	0.0677 (19)	
H12	0.1810	1.5863	0.4541	0.081*	
N4	0.3589 (8)	0.9870 (7)	−0.0535 (2)	0.0531 (12)	

### Atomic displacement parameters (Å^2^)

**Table:**

	*U*^11^	*U*^22^	*U*^33^	*U*^12^	*U*^13^	*U*^23^
Cd1	0.0446 (2)	0.03687 (19)	0.0315 (2)	−0.01765 (16)	0.00348 (18)	−0.00516 (15)
Cd2	0.0456 (3)	0.0426 (2)	0.0309 (2)	−0.01997 (17)	0.00095 (18)	−0.00324 (16)
S1	0.0417 (8)	0.0603 (8)	0.0370 (8)	−0.0204 (6)	0.0045 (6)	−0.0103 (6)
S3	0.0585 (10)	0.0841 (10)	0.0350 (8)	−0.0451 (8)	0.0040 (7)	−0.0085 (7)
S2	0.0482 (8)	0.0707 (9)	0.0340 (8)	−0.0345 (7)	0.0035 (6)	−0.0085 (6)
S4	0.0474 (9)	0.0867 (10)	0.0348 (8)	−0.0355 (8)	0.0032 (7)	−0.0087 (7)
N1	0.050 (3)	0.062 (3)	0.039 (3)	−0.030 (2)	0.005 (2)	−0.007 (2)
N3	0.053 (3)	0.068 (3)	0.036 (3)	−0.033 (2)	0.001 (2)	−0.007 (2)
N2	0.051 (3)	0.069 (3)	0.037 (3)	−0.031 (2)	−0.003 (2)	−0.004 (2)
N11	0.042 (3)	0.043 (2)	0.039 (3)	−0.0169 (19)	−0.001 (2)	−0.0019 (18)
N21	0.052 (3)	0.040 (2)	0.043 (3)	−0.020 (2)	0.000 (2)	−0.0059 (19)
N31	0.052 (3)	0.044 (2)	0.050 (3)	−0.020 (2)	0.001 (2)	−0.003 (2)
N41	0.050 (3)	0.043 (2)	0.056 (3)	−0.018 (2)	0.003 (3)	−0.002 (2)
C1	0.038 (3)	0.033 (2)	0.040 (3)	−0.018 (2)	−0.003 (2)	0.003 (2)
C3	0.037 (3)	0.037 (2)	0.037 (3)	−0.014 (2)	0.006 (2)	−0.002 (2)
C2	0.035 (3)	0.037 (2)	0.042 (3)	−0.016 (2)	0.009 (2)	−0.001 (2)
C4	0.040 (3)	0.039 (2)	0.039 (3)	−0.019 (2)	−0.008 (2)	−0.001 (2)
C11	0.060 (4)	0.049 (3)	0.047 (3)	−0.017 (3)	0.007 (3)	−0.004 (2)
C13	0.064 (4)	0.034 (3)	0.086 (5)	−0.014 (3)	−0.007 (4)	0.001 (3)
C14	0.069 (4)	0.048 (3)	0.066 (4)	−0.028 (3)	−0.006 (3)	0.014 (3)
C15	0.057 (4)	0.052 (3)	0.048 (4)	−0.021 (3)	0.004 (3)	0.002 (2)
C21	0.063 (4)	0.049 (3)	0.050 (4)	−0.024 (3)	0.007 (3)	−0.009 (3)
C22	0.067 (4)	0.051 (3)	0.069 (5)	−0.023 (3)	0.011 (3)	−0.023 (3)
C23	0.055 (4)	0.044 (3)	0.082 (5)	−0.016 (3)	−0.006 (3)	−0.013 (3)
C24	0.089 (5)	0.045 (3)	0.059 (4)	−0.027 (3)	−0.006 (4)	0.005 (3)
C25	0.072 (4)	0.050 (3)	0.043 (3)	−0.024 (3)	0.002 (3)	−0.005 (2)
C31	0.071 (4)	0.051 (3)	0.054 (4)	−0.024 (3)	−0.005 (3)	−0.006 (3)
C33	0.066 (4)	0.046 (3)	0.093 (6)	−0.023 (3)	0.001 (4)	−0.008 (4)
C34	0.081 (5)	0.052 (4)	0.073 (5)	−0.018 (3)	−0.009 (4)	0.015 (3)
C35	0.074 (4)	0.055 (4)	0.052 (4)	−0.021 (3)	−0.001 (3)	−0.001 (3)
C41	0.075 (4)	0.048 (3)	0.052 (4)	−0.018 (3)	0.004 (3)	−0.007 (3)
C42	0.080 (5)	0.063 (4)	0.072 (5)	−0.025 (4)	0.010 (4)	−0.025 (4)
C43	0.062 (4)	0.048 (3)	0.090 (6)	−0.024 (3)	0.005 (4)	0.002 (3)
C44	0.091 (6)	0.058 (4)	0.068 (5)	−0.029 (4)	−0.006 (4)	0.012 (3)
C45	0.074 (5)	0.052 (3)	0.057 (4)	−0.026 (3)	−0.008 (3)	0.001 (3)
C32	0.093 (6)	0.050 (4)	0.074 (5)	−0.027 (4)	0.006 (4)	−0.019 (3)
C12	0.076 (5)	0.046 (3)	0.064 (5)	−0.011 (3)	0.004 (4)	−0.011 (3)
N4	0.054 (3)	0.076 (3)	0.035 (3)	−0.035 (2)	0.007 (2)	−0.008 (2)

### Geometric parameters (Å, °)

**Table:**

Cd1—N3	2.298 (5)	C13—H13	0.9300
Cd1—N1^i^	2.317 (5)	C14—C15	1.378 (8)
Cd1—N11	2.365 (4)	C14—H14	0.9300
Cd1—N21	2.367 (4)	C15—H15	0.9300
Cd1—S2	2.7508 (15)	C21—C22	1.375 (8)
Cd1—S1	2.7715 (16)	C21—H21	0.9300
Cd2—N4^ii^	2.303 (5)	C22—C23	1.349 (10)
Cd2—N2	2.309 (5)	C22—H22	0.9300
Cd2—N41	2.363 (4)	C23—C24	1.361 (9)
Cd2—N31	2.366 (4)	C23—H23	0.9300
Cd2—S3	2.7416 (16)	C24—C25	1.385 (8)
Cd2—S4	2.7503 (17)	C24—H24	0.9300
S1—C1	1.647 (5)	C25—H25	0.9300
S3—C3	1.646 (6)	C31—C32	1.371 (9)
S2—C2	1.642 (6)	C31—H31	0.9300
S4—C4	1.643 (6)	C33—C32	1.360 (11)
N1—C1	1.156 (6)	C33—C34	1.365 (11)
N1—Cd1^i^	2.317 (5)	C33—H33	0.9300
N3—C3	1.144 (7)	C34—C35	1.371 (9)
N2—C2	1.159 (7)	C34—H34	0.9300
N11—C11	1.329 (7)	C35—H35	0.9300
N11—C15	1.342 (7)	C41—C42	1.379 (8)
N21—C21	1.326 (7)	C41—H41	0.9300
N21—C25	1.332 (7)	C42—C43	1.358 (11)
N31—C35	1.318 (8)	C42—H42	0.9300
N31—C31	1.329 (8)	C43—C44	1.360 (11)
N41—C45	1.324 (8)	C43—H43	0.9300
N41—C41	1.331 (8)	C44—C45	1.370 (9)
C4—N4	1.153 (7)	C44—H44	0.9300
C11—C12	1.378 (8)	C45—H45	0.9300
C11—H11	0.9300	C32—H32	0.9300
C13—C14	1.357 (10)	C12—H12	0.9300
C13—C12	1.360 (10)	N4—Cd2^ii^	2.303 (5)
			
N3—Cd1—N1^i^	95.37 (17)	C14—C13—H13	120.6
N3—Cd1—N11	90.26 (17)	C12—C13—H13	120.6
N1^i^—Cd1—N11	91.19 (16)	C13—C14—C15	119.2 (6)
N3—Cd1—N21	90.51 (17)	C13—C14—H14	120.4
N1^i^—Cd1—N21	91.07 (16)	C15—C14—H14	120.4
N11—Cd1—N21	177.54 (15)	N11—C15—C14	122.9 (6)
N3—Cd1—S2	92.60 (12)	N11—C15—H15	118.6
N1^i^—Cd1—S2	172.02 (13)	C14—C15—H15	118.6
N11—Cd1—S2	88.36 (11)	N21—C21—C22	123.3 (6)
N21—Cd1—S2	89.27 (12)	N21—C21—H21	118.3
N3—Cd1—S1	175.47 (12)	C22—C21—H21	118.3
N1^i^—Cd1—S1	89.01 (13)	C23—C22—C21	119.1 (6)
N11—Cd1—S1	90.83 (12)	C23—C22—H22	120.4
N21—Cd1—S1	88.23 (12)	C21—C22—H22	120.4
S2—Cd1—S1	83.03 (4)	C22—C23—C24	119.3 (6)
N4^ii^—Cd2—N2	94.30 (17)	C22—C23—H23	120.3
N4^ii^—Cd2—N41	91.31 (18)	C24—C23—H23	120.3
N2—Cd2—N41	91.26 (18)	C23—C24—C25	118.4 (6)
N4^ii^—Cd2—N31	91.68 (17)	C23—C24—H24	120.8
N2—Cd2—N31	90.34 (17)	C25—C24—H24	120.8
N41—Cd2—N31	176.50 (16)	N21—C25—C24	123.2 (6)
N4^ii^—Cd2—S3	174.04 (13)	N21—C25—H25	118.4
N2—Cd2—S3	91.52 (13)	C24—C25—H25	118.4
N41—Cd2—S3	87.27 (13)	N31—C31—C32	122.4 (7)
N31—Cd2—S3	89.57 (12)	N31—C31—H31	118.8
N4^ii^—Cd2—S4	92.06 (14)	C32—C31—H31	118.8
N2—Cd2—S4	173.51 (13)	C32—C33—C34	118.1 (6)
N41—Cd2—S4	89.88 (14)	C32—C33—H33	120.9
N31—Cd2—S4	88.19 (13)	C34—C33—H33	120.9
S3—Cd2—S4	82.16 (5)	C33—C34—C35	118.8 (7)
C1—S1—Cd1	103.53 (19)	C33—C34—H34	120.6
C3—S3—Cd2	101.98 (18)	C35—C34—H34	120.6
C2—S2—Cd1	101.04 (17)	N31—C35—C34	123.6 (6)
C4—S4—Cd2	101.36 (19)	N31—C35—H35	118.2
C1—N1—Cd1^i^	152.4 (4)	C34—C35—H35	118.2
C3—N3—Cd1	161.5 (4)	N41—C41—C42	122.6 (6)
C2—N2—Cd2	163.6 (4)	N41—C41—H41	118.7
C11—N11—C15	116.7 (5)	C42—C41—H41	118.7
C11—N11—Cd1	122.2 (4)	C43—C42—C41	119.1 (7)
C15—N11—Cd1	121.1 (4)	C43—C42—H42	120.5
C21—N21—C25	116.7 (5)	C41—C42—H42	120.5
C21—N21—Cd1	123.0 (4)	C42—C43—C44	119.0 (6)
C25—N21—Cd1	120.3 (4)	C42—C43—H43	120.5
C35—N31—C31	117.2 (5)	C44—C43—H43	120.5
C35—N31—Cd2	121.2 (4)	C43—C44—C45	118.7 (7)
C31—N31—Cd2	121.4 (4)	C43—C44—H44	120.7
C45—N41—C41	117.1 (5)	C45—C44—H44	120.7
C45—N41—Cd2	121.6 (4)	N41—C45—C44	123.6 (7)
C41—N41—Cd2	121.3 (4)	N41—C45—H45	118.2
N1—C1—S1	178.5 (4)	C44—C45—H45	118.2
N3—C3—S3	179.1 (5)	C33—C32—C31	119.8 (7)
N2—C2—S2	179.7 (5)	C33—C32—H32	120.1
N4—C4—S4	178.8 (5)	C31—C32—H32	120.1
N11—C11—C12	123.2 (6)	C13—C12—C11	119.2 (6)
N11—C11—H11	118.4	C13—C12—H12	120.4
C12—C11—H11	118.4	C11—C12—H12	120.4
C14—C13—C12	118.8 (5)	C4—N4—Cd2^ii^	162.1 (4)

Symmetry codes: (i) −*x*+1, −*y*+2, −*z*+1; (ii) −*x*+1, −*y*+2, −*z*.
